# Don't lose sight of the importance of the individual in effective falls prevention interventions

**DOI:** 10.1186/1471-2318-9-13

**Published:** 2009-04-22

**Authors:** Keith Hill

**Affiliations:** 1 Musculoskeletal Research Centre, Faculty of Health Sciences, La Trobe University and Northern Health, Bundoora, Victoria, 3086 Australia; 2 Preventive and Public Health Division, National Ageing Research Institute, Poplar Rd, Parkville, Victoria, 3052 Australia

## Abstract

Falls remain a major public health problem, despite strong growth in the research evidence of effective single and multifactorial interventions, particularly in the community setting. A number of aspects of falls prevention require individual tailoring, despite limitations being reported regarding some of these, including questions being raised regarding the role of falls risk screening and falls risk assessment. Being able to personalise an individual's specific risk and risk factors, increase their understanding of what interventions are likely to be effective, and exploring options of choice and preference, can all impact upon whether or not an individual undertakes and sustains participation in one or more recommendations, which will ultimately influence outcomes. On all of these fronts, the individual patient receiving appropriate and targeted interventions that are meaningful, feasible and that they are motivated to implement, remains central to effective translation of falls prevention research evidence into practice.

## 

Falls and associated injuries remain a major public health problem, with little evidence that deaths and serious injuries from falls are declining [1-3], despite a strong focus in research and practice over the past 15 years. While research has demonstrated that many single interventions and multi-factorial interventions can be effective [4,5], the effective translation of these approaches into practice has generally been mixed. For example, one study has highlighted that evidence based practice occurred for 4% of older people presenting to Emergency Departments after a fall [6]. More promising translation was demonstrated in a recent project in the United States, where evidence based interventions with a practice change focus were supported in a region of Connecticut, and achieved a significant reduction in falls and fall related medical service use compared to a nearby control region [7]. While a strong focus of the research has been on the nature and scope of interventions, a critical aspect that has tended to be marginal in its focus is the role of the older person in successful falls prevention.

An important aspect of individualising falls prevention interventions is the use of falls risk screening and falls risk assessment. Falls risk among older people varies along a continuum, from those who are healthy and active, through to those with high level of frailty, multiple comorbidities, and high falls and injury risk. Falls can occur at any point along this continuum, however much of the falls prevention research has targeted the frail end of the spectrum. In one study of well screened healthy women aged over 70 followed prospectively over 12 months, 49% fell, and 9% had a fracture as a result of a fall [8]. Similarly, Speechley and Tinetti [9] identified high injury rates from falls among "vigorous" older people. Falls among this group tend to be much more commonly due to environment, lack of concentration, and multi-tasking activities, rather than easily predictable or identifiable intrinsic risk factors. From an early falls risk management perspective, identifying risk at a mild level, and intervening at this early stage, has potential to achieve greater impact than waiting for falls risk to become more advanced before engagement with the health service system. This an area warranting research focus.

There has been growing criticism of falls risk screening and assessment tools recently, in particular related to the limited prediction accuracy of these tools [10,11]. Falls risk screening involves a brief evaluation (usually less than 5 items) that classifies a person's risk of falls, but does not provide a basis to plan an individualised treatment plan, because of the general nature and small number of items reviewed. Probably the best screening item in isolation is history of previous falls, which has consistently been shown to be a strong risk factor for falls [12], and is a component of the majority of community based falls risk screening tools (eg [13,14]). The purpose of a screen is to determine those who exceed the threshold risk level, an indication of need for a full falls risk assessment.

Falls risk assessment can involve a detailed structured assessment, or can involve use of a falls risk assessment tool. The main purpose of a falls risk assessment tool is not to predict falls risk, but to identify presence of contributory factors to the individual's falls risk, which can then form the basis of a multi-factorial falls prevention intervention. Falls risk on individual risk factors can vary in severity, and even presence of mild levels of risk on an individual risk factor should be considered for intervention, from a preventive perspective. Relatively few falls risk assessments provide graded risk on individual risk factors. Examples that do are the Physiological Profile Assessment [15] and the FROP-Com [16].

Without using falls risk screening tools or falls risk assessment tools, the only approach to introducing multi-factorial interventions is to introduce falls prevention actions universally, perhaps to all identified as at increased risk. This approach has the potential to be applied to many people who do not specifically need the intervention, and to not provide required interventions for others. Identifying risk and risk factors is very important to efficient targeting of falls prevention interventions. Application of a comprehensive falls risk assessment [17,18] can be the basis for effective falls prevention for individuals, particularly those at increased risk.

A potential factor limiting effectiveness of falls prevention activities is low levels of uptake and sustained engagement in recommended falls prevention activities by the older individual. Improving knowledge among older people, health professionals and carers and other staff involved with older people – that evidence based interventions can reduce falls – is likely to improve engagement with recommendations [19]. Involvement of the older individual and their families in discussing their risk factors, goal setting, and preferences, and in linking falls prevention messages to messages promoting maintenance of independence and function may also result in improved uptake and adherence [20].

Frail older people often have many falls risk factors. For example, people attending a specialist falls clinics in Australia each received an average of six new falls prevention recommendations [21]. There may be a case for prioritising interventions, and possibly selecting a limited number of interventions to address initially, to maximise engagement and minimise confusion and fatigue. Older people need to perceive their health problem of falls as of high importance relative to other co-morbidities in order to be likely to implement recommended interventions [22]. Furthermore, a recent publication recommended that a small number of targeted interventions might be as effective, and more cost effective, than utilising a multiple factor falls prevention program [23], although further research is needed comparing single and multiple intervention approaches.

Older people with mild levels of falls risk are often not considered to be at risk, because they appear reasonably active and mobile. However, an example where mild levels of falls risk is often ignored is where an older person feels unsteady or feels their balance or mobility is not as good as it was previously. Many older people with these concerns who report them to a health practitioner are told "what do you expect at 75 years, you've got to expect some unsteadiness or falls...". By giving this response, the health practitioner is not considering the potential for the problem to be more than just the effect of age, but instead some developing health problems impacting upon balance and mobility that may be potentially remediable. In a recent study by our team, Yang et al [24] investigated community ambulant older people with concerns about their balance or mobility, and identified that almost three quarters had an identifiable balance problem relative to normative performance. If a home exercise approach can be shown to be effective at this early stage of balance impairment, this may prevent some of these people progressing to more advanced risk of falls.

Another way that falls prevention actions can be implemented at an earlier stage of the falls risk continuum is to support improved identification of older people who have had a fall. Over two thirds of older people who fall may not report the fall to health professionals or their families [8,25], particularly if the fall does not cause an injury. Given the strong evidence that previous falls, even those that do not cause an injury, are a strong predictor of future falls, a key falls prevention target needs to be improving identification of people who fall. Best practice guidelines recommend that all general practitioners should ask all older patients at least once each year whether they have had a fall [26]. If the individual patient answers yes, they should then undergo an assessment to determine whether modifiable falls risk factors are present. The other avenue to increase reporting is to promote this information broadly to older people through a range of promotional avenues, including use of self checklists of falls risk, that could be used in general practitioners' offices, or distribution through home care workers. The key messages of these documents needs to be that falls are not just due to age, that many falls can be prevented, and some simple questions about presence of common falls risk factors that indicate the need for a health practitioner review.

Falls risk can vary over time for the one individual. In particular, acute health problems, and hospitalisation increases risk of falling. Falls rates are high for older people in the month after discharge home, with 15% falling at least once, and 11% of these requiring re-admission to hospital [27]. Some clinical groups also have high falls risk following discharge home from hospital, one of the highest risk groups is people being discharged home from hospital after stroke. In one study, 46% of stroke patients fell in the six months following discharge from hospital, with 42% of these occurring in the first month [25]. Hospital discharge planning needs to have a stronger focus on ensuring falls prevention is a core element post discharge.

The older individual, their individual mix of falls risk factors, their preferences in terms of interventions, and their engagement in recommended interventions in a sustained manner are all essential to effective falls prevention. These factors require a stronger focus in falls prevention research and practice.

## Competing interests

The author declares that they have no competing interests.

## About the author

Keith Hill is Professor of Allied Health at La Trobe University, Northern Health, and the National Ageing Research Institute. He is a physiotherapist with experience in falls prevention research in community, hospital and residential care settings.

## Pre-publication history

The pre-publication history for this paper can be accessed here:


